# smAvo and smaTo: A fruity odyssey of smart sensor platforms in Southern Africa

**DOI:** 10.1016/j.ohx.2020.e00156

**Published:** 2020-11-04

**Authors:** André Broekman, Wynand JvdM Steyn, Johannes LP Steyn, Malick Bill, Lise Korsten

**Affiliations:** a Department of Civil Engineering, University of Pretoria, Pretoria, South Africa; b Department of Electrical, Electronic and Computer Engineering, University of Pretoria, South Africa; c Department of Plant and Soil Sciences, University of Pretoria, South Africa

**Keywords:** Civiltronics, Smart fruit, Sensor platform, Postharvest, Avocadoes, Tomatoes, Agricultural engineering, Transportation engineering

## Abstract

Avocado and tomato production are key agricultural sectors for many economies including South Africa. The quality of avocadoes and tomatoes that reach consumers is directly dependent on the handling at the postharvest stage. The fruit undergoes an extensive journey, and subsequently stress, comprised of several steps, including harvesting, application of postharvest treatments, packaging and transportation by road and sea to reach the export markets. To date, the quantification of these external stresses on a discrete fruit has not been measured in detail, primarily due to a lack of compatible instrumentation. This paper demonstrates a low cost, stand-alone, open source data acquisition system, termed smAvo and smaTo, that can be introduced effortlessly to monitor agricultural processing facilities and transportation networks. The miniaturised Arduino sensor platform is enclosed within a waterproof enclosure and surrounded by a 3D printed shell manufactured from morphologically compatible materials. The software is customisable to the needs of the research project or individual transportation phases under study, providing both extensive environmental data and high-frequency tri-axis acceleration measurements that are crucial to understanding the dynamic processes that directly affect the final quality of the fruit. Four successful field trials demonstrate the fidelity of both the smAvo and smaTo platforms.

## Highlights

• Sensor platform measuring mesoscale particle behaviour.
• Sensor platform for smart agricultural engineering applications.
• Data-driven postharvest optimisation through quantitative measurements.
• Quantifying stress of avocadoes and tomatoes using smAvo and smaTo.

## Graphical abstract

**Figure:**
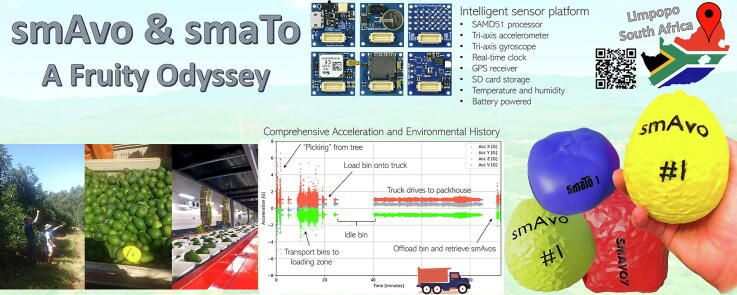

Specifications table

**Table:** 

Hardware name	smAvo and smaTo
Subject area	Agricultural Sciences
Hardware type	Field measurements and sensors
Open Source License	Creative Commons Attribution-ShareAlike
Cost of Hardware	$210.14 (smAvo) $191.55 (smaTo)
Source File Repository	https://doi.org/10.17605/OSF.IO/3H74M

## 1 Hardware in context

The South African avocado market ranked as the 12th largest globally for 2019, exporting over 43,000 tons of avocadoes [1] amounting to $60 million. Comparatively, the gross value of local tomato production amounted to $230 million in 2016 [2]. Every individual fruit forms part of a vast and complex supply chain, moving from the field to the packhouse for sorting, grading, stacking, cooling and loading, through to the market and consumer. The transportation of avocadoes is comprised of various modes, including road and sea, which requires specific environmental and performance conditions to be maintained within a narrow range (e.g. temperature, relative humidity and volatile gasses). This minimisation of external stresses on the fruit, both environmental and physical, correlates directly to the quality and appearance received by the consumer. The management of the fruit quality at the postharvest stage and during the supply chain is one of the primary drivers in ensuring profitability whilst reducing food losses and wastes [3].

**Fig. 1:**
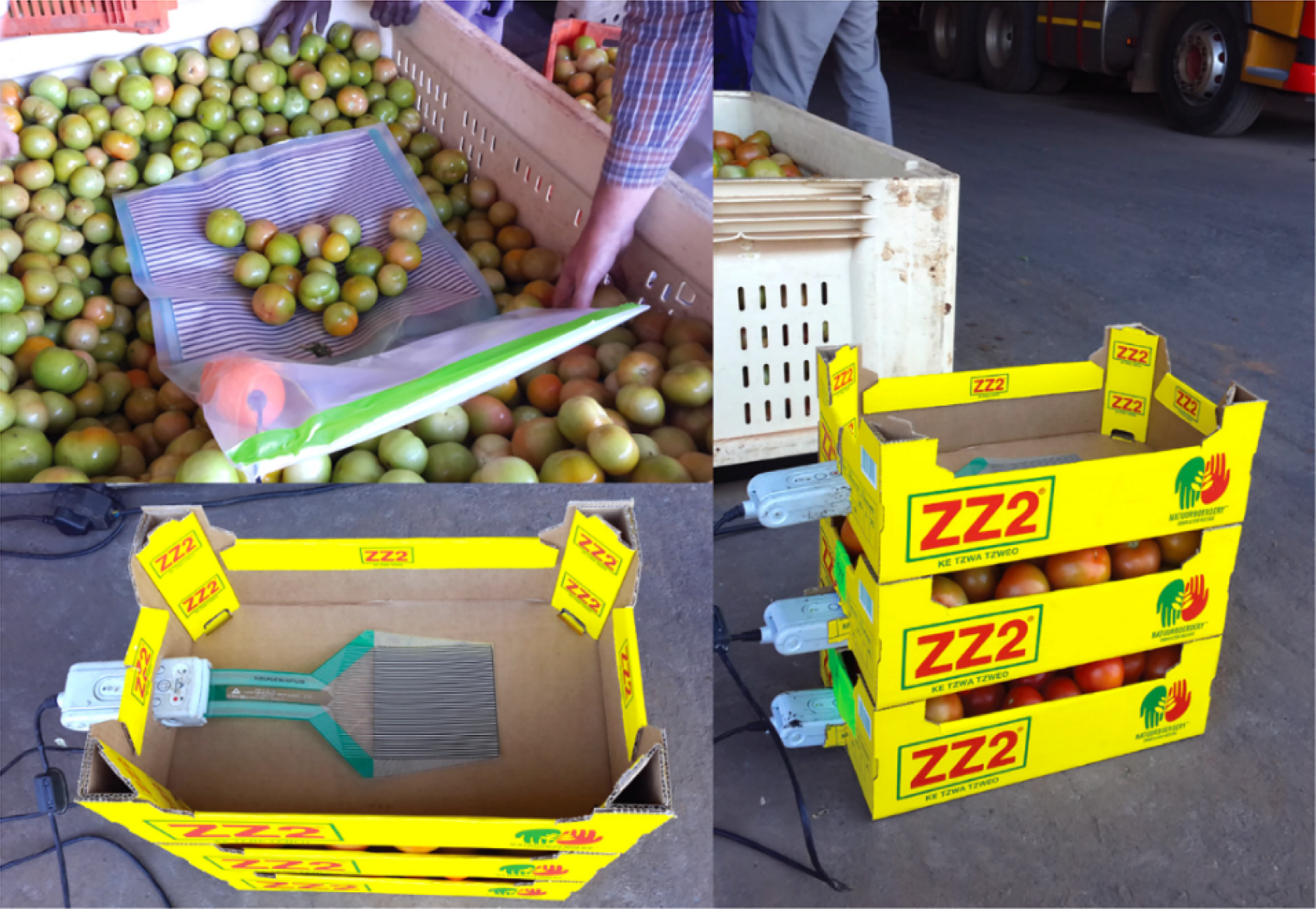
Pressure sensor matrix for measuring interfacial tomato stresses during transit (from [11]).

Extensive field trials of tomatoes have demonstrated the influence of pavement riding quality on both the logistical costs [4] and the quality of the fresh produce [5]. Whilst desktop studies are useful for providing as estimate of cost-to-benefit for remedial measures [6], quantification of riding quality – the primary measure of potential damage or induced stress for fresh produce transported by road – ultimately provide the necessary detailed investigation to calibrate and validate new and existing models. New technology, such as vehicle tracking devices [7], [8], seek to address the requirement for larger datasets, specifically in rural areas [9] where monitoring transportation systems accurately proves difficult. Acceleration and interfacial stress measurements of tomatoes during transportations have shown a clear influence on both the postharvest quality of the fruit and shelf life [10], [11], [12]. These accelerations were measured using commercial tri-axis accelerometer devices with the stresses quantified by flexible pressure mats containing a square matrix of discrete stress measurement points. These accelerometers were installed along various locations in the vehicle, with the pressure mats positioned along both the bottom of smaller boxes and in the middle of larger bins (Fig. 1).

Whilst these solutions provide highly accurate data at a suitable frequency, significant human resources and installation time is required for field measurements. Additionally, instruments are dependent on external power sources, increasing both the complexity and possible points of failure. Systematic errors are present as the fruits cannot be *directly* instrumented on a discrete or mesoscale. With the proliferation of low-cost microcontroller and microcomputer solutions in the preceding decade, new avenues for developing customised instrumentation, specifically for agricultural research, became widely available. Examples include the monitoring of air quality [13] and plant growth [14] using Raspberry Pis, numerous and varied applications of Arduino microcontrollers on farms [15] and sensor platforms in the form of a potato (*TuberLog*) [16]. The intersection between open source hardware solutions and the need for instrumenting fresh produce lies in the development of a digital twin sensor platform. This approach has been successfully applied in the area of railway engineering with the development of Kli-Pi, a stand-alone, pseudo-ballast sensor platform that can be directly integrated into the matrix of particles that are under investigation [17], [18]. The fusion of instrumentation and synthetic samples present the ideal solution for investigating mesoscale phenomena of a particle that contributes to the macroscopic behaviour of the system as a whole.

The ongoing development and research of developing digital twin systems for agricultural application stems from both a need to optimize the postharvest stress experienced by fresh produce and to enhance the traceability and accountability of the supply chain. Cost efficient digital twin-driven joint optimization [19] and manufacturing cyber-physical system (MCPS) [20] frameworks can readily be adapted for real-time optimization leveraging IIoT (industrial internet of things) devices [21], reducing latency with the introduction of online (real-time communication) sensor platforms. Concerns currently experienced by the local agricultural industry, in particular theft of produce (lacking any unique identifiers) prior to harvesting, can benefit from blockchain-driven smarts contracts designed around hybrid QR-codes with unique chemical signatures [22]. Blockchain technologies can extend beyond the boundaries of the local packhouse and regional markets, addressing the need for more effective lifecycle management [23] associated with interlinked, complex transportation chains stretching across international borders and different modes of transportation.

This paper proposes an open-source, sensor platform for agricultural research applications that is morphologically compatible with the fruit under study. The miniaturised electronics are sealed off in a waterproof enclosure and surrounded by a 3D printed shell, replicating the elasticity and surface contact properties of its biological twin. These stand-alone sensor platforms, termed smAvo and smaTo for tomato and avocado fruits, respectively, can be introduced and removed at any point along the transportation and processing chain. These sensor platforms are programmed according to the project specifications, providing high-frequency acceleration and environmental data of the entire journey, by road and/or sea. smAvo and smaTo avoids labour intensive instrumentation installation procedures and do not require special considerations or modifications of workflows during experiments. The introduction of 3D printing of the external shells accelerates the research and development phases of the project in addition to producing near identical instruments for use in research applications. This project is an evolution of the concept of Civiltronics [24], leveraging affordable technological solutions to address research requirements in the era of the fourth industrial revolution, of which Transportation Engineering forms a key discipline [25], [26].

## 2 Hardware description

Based on prior research experience and new knowledge acquired with the introduction of smart instrumentation within the Department of Civil Engineering at the University of Pretoria, South Africa, a new sensor platform was proposed that could address the demanding research requirements and extreme testing requirements. The aim of the instrumentation is not necessarily to displace the use of specialised commercial hardware solutions. Instead, it addresses the research requirements from a “deemed to satisfy” perspective, where existing, low-cost hardware solutions can be adopted. The unique requirements to monitor discrete fruits to continuously monitor environmental and physical stresses, necessitates the adoption of a completely new class of instrumentation. The following instrumentation requirements were established for the project:

1. Stand-alone sensor platform powered from a high capacity, rechargeable battery;
2. Sufficient run-time for localised testing (4 h continuous) with the option to extend to inter-provincial travel (3 days intermittent);
3. High-frequency, MEMS-based tri-axis accelerometer;
4. Integrated GPS for monitoring geolocation data;
5. Programmable firmware alongside high-capacity, non-volatile storage;
6. Small physical footprint that conforms to typical Hass avocado sizes;
7. Waterproof for short periods of time (during packhouse processing);
8. An easily removable, protective, 3D printed shell that can interlock during testing, and
9. Simple to operate and train researchers to use the instrument.

From the available hardware solutions, the TinyDuino sensor platform developed and manufactured by TinyCircuits (located in the United States of America) was selected [27]. The TinyDuino is based on the Arduino family of microcontrollers and integrates with the existing programming IDE and software development pipeline. The TinyDuino addresses both the hardware and software requirements whilst retaining the flexibility to program the sensor platform for the desired application. The use of vertically aligned shields into a compact “stack” substantially minimises the volume occupied by the electronics (Fig. 3). Six boards were selected to address all the requirements of the project:

1. TinyZero processor board (ASM2021-R-A);
2. MicroSD TinyShield (ASD2201-R);
3. TinyShield proto board (ASD2009-R-T);
4. Real-time clock (RTC) TinyShield (ASD2831-R);
5. Combo sensor TinyShield (ASD2511), and
6. GPS TinyShield (ASD2501-R).

**Fig. 2:**
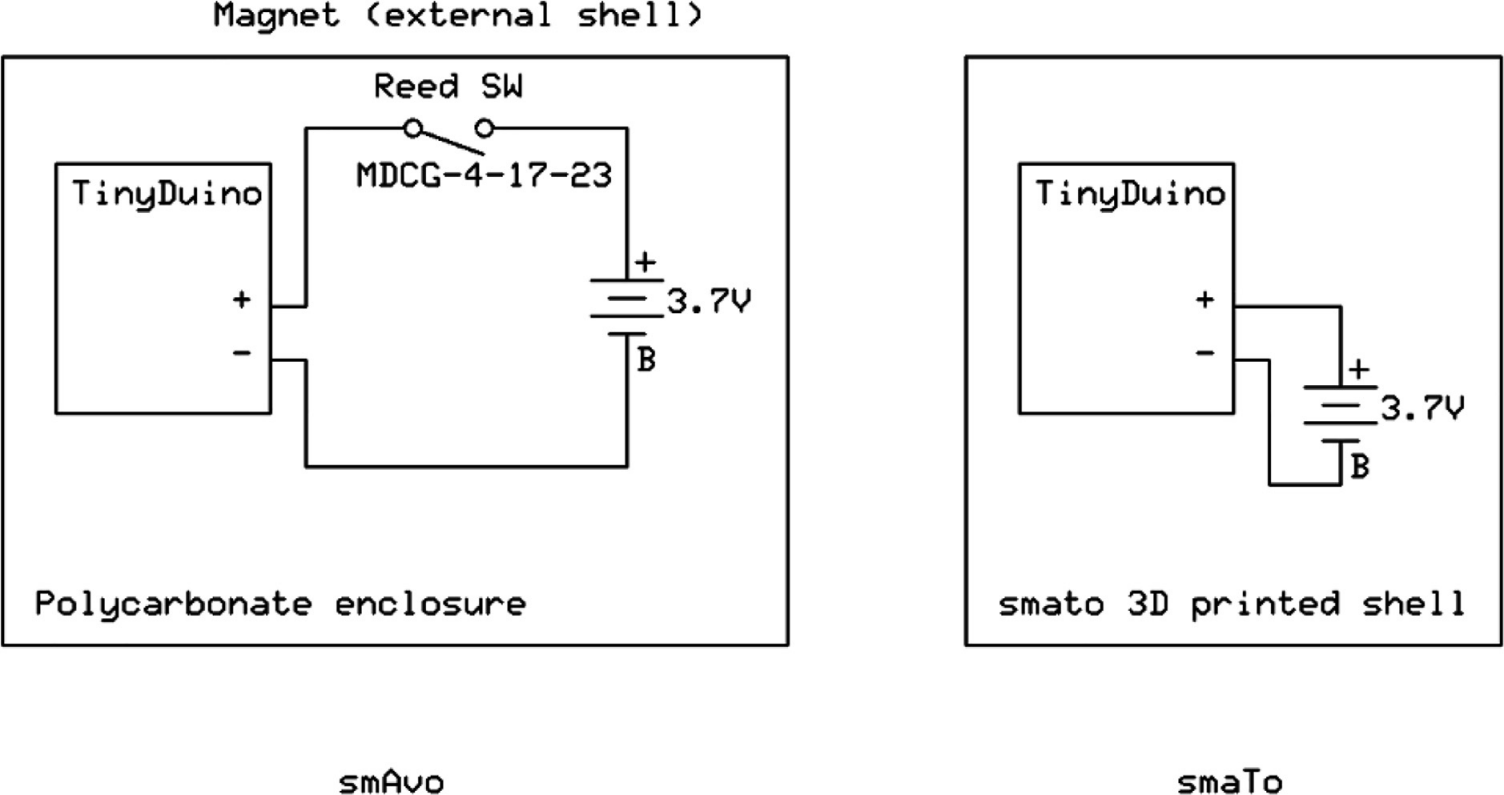
Electronic schematic of the final smAvo and smaTo prototypes.

Each of the shields measured 20 mm on each side with the total stack height measuring approximately 30 mm tall. The stack is fixed together using the included mounting kit. The battery is pre-configured with the required JST-SH but can be soldered onto other compatible LiPo cells with different dimensions and capacities. The schematic of the final prototypes of both smAvo and smaTo are illustrated in Fig. 2. A detailed discussion follows of the design and development of the prototypes in parallel with the implementation of 3D printing for the protective shell.

The Fly-by-Pi controller demonstrates the following advantages that is applicable to the wider user community:

1. The miniaturisation of the sensor platform (TinyDuino) enables new research avenues, specifically for environmental and animal studies where traditional instrumentation is too large and obtrusive in natural habitats. For example, measuring the temperature and relative humidity within small burrows;
2. The modularity of the Arduino platform accommodates 3rd party sensors and expansion boards without the need for hardware or software modification, and
3. The solid-state design of the electronics provides immunity to mechanical and thermal shocks. To date, no hardware failure has been experienced apart from direct and prolonged contact with water.

## 3 Design files

The complete list of files is summarised in Table 1. These files provide the necessary information and software to duplicate and implement an equivalent smAvo or smaTo sensor platform. The files are freely accessible at the Open Science Framework file repository linked together with this manuscript.Table 2

**Table 1:** Complete list of design files.

Design file name	File type	Open source license	Location of the file
smAvo_smaTo.pdf	PDF	CC BY 4.0	Source file repository (Electronics Design folder)
smAvo_smaTo.sch	ExpressSCH	CC BY 4.0	Source file repository (Electronics Design folder)
1_Freeway.zip	Zip (CSV)	CC BY 4.0	Source file repository (Example Data folder)
2_Paved_Unpaved_Road.zip	Zip (CSV)	CC BY 4.0	Source file repository (Example Data folder)
3_smAvo_Field_Test.zip	Zip (CSV + Media)	CC BY 4.0	Source file repository (Example Data folder)
4_smAvo_Packhouse.zip	Zip (CSV + Media)	CC BY 4.0	Source file repository (Example Data folder)
5_smaTo_Packhouse.zip	Zip (CSV + Media)	CC BY 4.0	Source file repository (Example Data folder)
Testing_Procedure_Checklist.pdf	PDF	CC BY 4.0	Source file repository (Example Data folder)
30_second_100Hz.zip	Zip (Arduino sketch)	CC BY 4.0	Source file repository (Firmware folder)
Continuous_100Hz.zip	Zip (Arduino sketch)	CC BY 4.0	Source file repository (Firmware folder)
Libraries.zip	Zip (Arduino libraries)	CC BY 4.0	Source file repository (Firmware folder)
Sensor_Documetation.zip	Zip (PDF)	CC BY 4.0	Source file repository (Firmware folder)
smAvo.zip	Zip (STL and Blender)	CC BY 4.0	Source file repository (Models folder)
smaTo.zip	Zip (STL and Blender)	CC BY 4.0	Source file repository (Models folder)

**Table 2:** Sensor platform bill of materials.

Designator	Component	Number	Cost per unit - currency	Total cost - currency	Source of materials	Material type
TinyDuino	TinyZero processor board (ASM2021-R-A, accelerometer present)	1	$24.95 USD	$29.95 USD	TinyCircuits	Other
	MicroSD TinyShield (ASD2201-R)	1	$14.95 USD	$14.95 USD	TinyCircuits	Other
	TinyShield proto board (ASD2009-R-T, connector present)	1	$3.95 USD	$3.95 USD	TinyCircuits	Other
	Real-time clock TinyShield (DS1339 | ASD2831-R)	1	$19.95 USD	$19.95 USD	TinyCircuits	Other
	Combo sensor TinyShield (9-Axis IMU, temperature, humidity, pressure, light | ASD2511)	1	$39.95 USD	$39.95 USD	TinyCircuits	Other
	GPS TinyShield (Telit JF2 GPS Module | ASD2501-R)	1	$59.95 USD	$59.95 USD	TinyCircuits	Other
	Battery (Lithium ion polymer 3.7 V 1100mAh | ASR00008)	1	$9.95 USD	$9.95 USD	TinyCircuits	Other
	SD Card (8 Gb Micro SD)	1	$9.95 USD	$9.95 USD	TinyCircuits	Other
	TinyDuino mounting hardware kit Details (ASH1002)	1	$3.95 USD	$3.95 USD	TinyCircuits	Other
Sensor platform	Total cost per sensor platform			$187.55 USD		

*smAvo_smaTo.pdf*: Electronic schematic file for both smAvo and smaTo (identical to Fig. 2).

*smAvo_smaTo.pdf*: ExpressSCH electronic schematic file for both smAvo and smaTo (identical to Fig. 2).

*1_Freeway.zip*: Collection of field data (refer to Section 7.1). This includes 30-second smAvo accelerometer data (CSV) and an environmental data log file (CSV) of a trip on a freeway.

*2_Paved_Unpaved_Road.zip*: Collection of field data (refer to Section 7.1). This includes 30-second smAvo accelerometer data (CSV) and an environmental data log file (CSV) of data recorded for both a paved and unpaved road section.

*3_smAvo_Field_Trip.zip*: Collection of field data (refer to Section 7.2). This includes 30-second smAvo accelerometer data (CSV) and an environmental data log file (CSV) of three smAvos from the field to the packhouse. Includes additional photographs and a video of on the farm as well as a variety of statistical graphs generated using post-processing tools.

*4_smAvo_Packhouse.zip*: Collection of field data (refer to Section 7.3). This includes 30-second smAvo accelerometer data (CSV) and an environmental data log file (CSV) from one smAvo measuring the packhouse process. Includes a variety of statistical graphs generated using post-processing tools.

*5_smaTo_Packhouse.zip*: Collection of field data (refer to Section 7.4). This includes 30-second smaTo accelerometer data (CSV) and an environmental data log file (CSV) from one smaTo measuring the packhouse process. Includes a variety of statistical graphs generated using post-processing tools.

*Testing_Procedure_Checklist.pdf*: A standardised testing procedure and checklist used during for acquiring data from packhouses for both the smaTos and smAvos.

*30_second_100Hz.zip*: Arduino sketch for recording 100 Hz tri-axis acceleration data in 30 s segments (refer to Section 6.1) together with the libraries required for the software serial interface.

*Continuous_100Hz.zip*: Arduino sketch for recording 100 Hz tri-axis acceleration data continuously (refer to Section 6.1) together with the libraries required for the software serial interface.

*Libraries.zip*: Arduino libraries for all the TinyDuino sensors, SD card interface and GPS as required by the above mentioned Arduino sketches.

*Sensor_Documentation.zip*: Detailed sensor documentation for the TinyDuino sensors (PDFs).

*smAvo.zip*: STL and Blender files to replicate the smAvo shell (current iteration; refer to Section).

*smaTo.zip*: STL and Blender files to replicate the smaTo shell (current iteration; refer to Section).

## 4 Bill of materials

The complete bill of materials (BOM) to replicate both the smAvo (third prototype) and smaTo is listed in Table 3 and Table 4 respectively. For brevity, only the current implementation of the smAvo (third prototype) and smaTo is included in the BOM. Table 2 summarises the stand-alone TinyDuino sensor platform that is incorporated into both the smAvo and smaTo. The cost of shipping is not included in the price as this is highly dependent on the destination country, exchange rates and number of instruments required.

**Table 3:** smAvo bill of materials.

Designator	Component	Number	Cost per unit – currency	Total cost – currency	Source of materials	Material type
Enclosure	Enclosure (Fibox polycarbonate enclosure, IP67, 65 × 50 × 45 mm, 2207231)	1	$9.75 USD	$9.75 USD	RS	Polymer
Reed switch	Reed switch (pack of 5, Hamlin MDCG-4–17-23, 9058881)	1	$3.19 USD	$3.19 USD	RS	Other
Magnet	Magnet (N35-grade, 5 mm diameter)	1	$2.25 USD	$2.25 USD	RS	Metal
Shell	3D filament (TPU)	1	$7.40 USD	$7.40 USD	3dprintingstore	Polymer
Sensor platform	Total cost per sensor platform	1		$187.55 USD		
smAvo	Total cost per smAvo			$210.14 USD

**Table 4:** smaTo bill of materials.

Designator	Component	Number	Cost per unit – currency	Total cost – currency	Source of materials	Material type
Shell	3D filament (TPU)	1	$4.00 USD	$4.00 USD	3dprintingstore	Polymer
Sensor platform	Total cost per sensor platform	1	$187.55 USD	$187.55 USD		
smaTo	Total cost per smaTo			$191.55 USD		

## 5 Build instructions

The build instructions highlight the first two smAvo prototypes that were trailed and iteratively improved which lead to the current implementation of both the smAvo and smaTo. Considerations for different filament materials are also provided alongside optimised printer settings for each.

### 5.1 smAvo

**Fig. 3:**
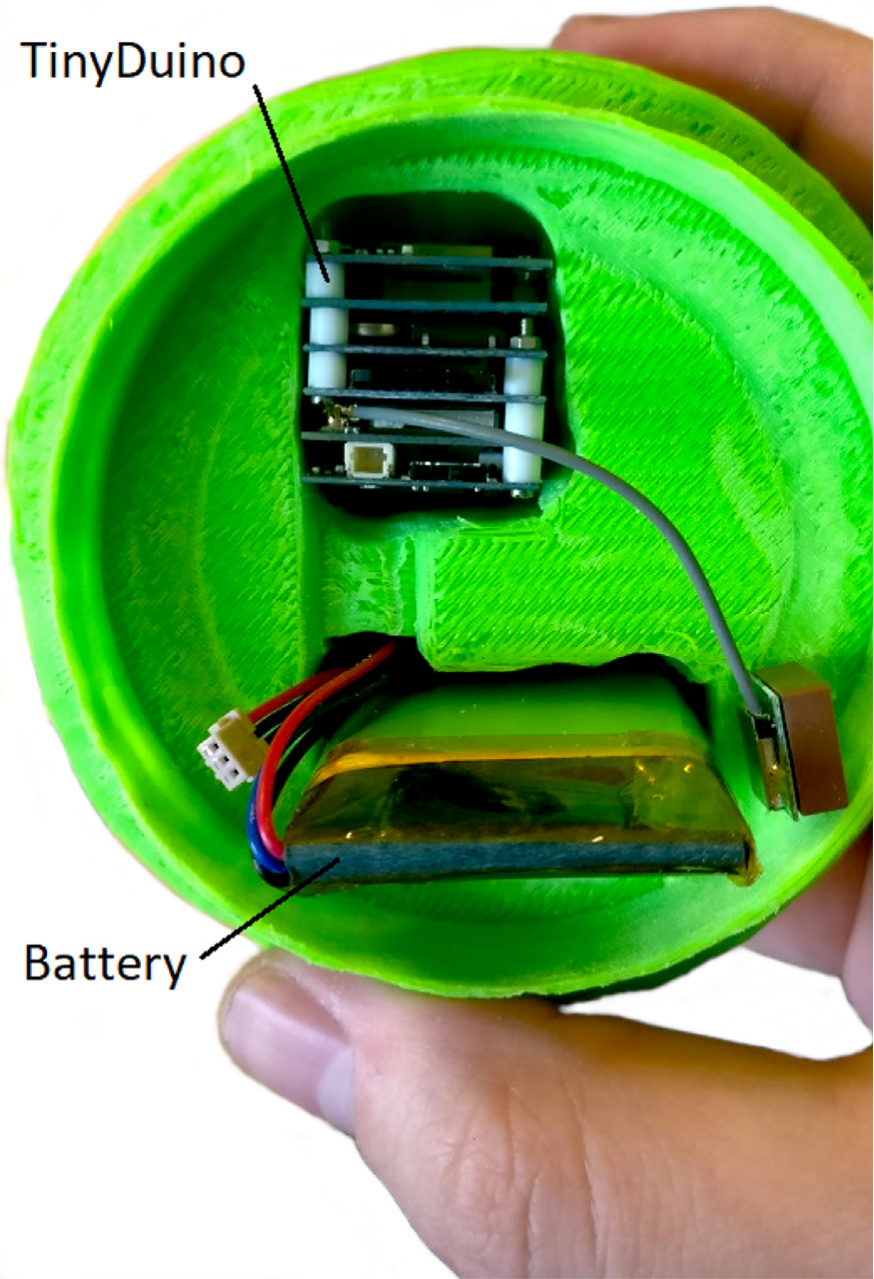
First prototype illustrating the TinyDuino stack and battery inside of the 3D test print.

**Fig. 4:**
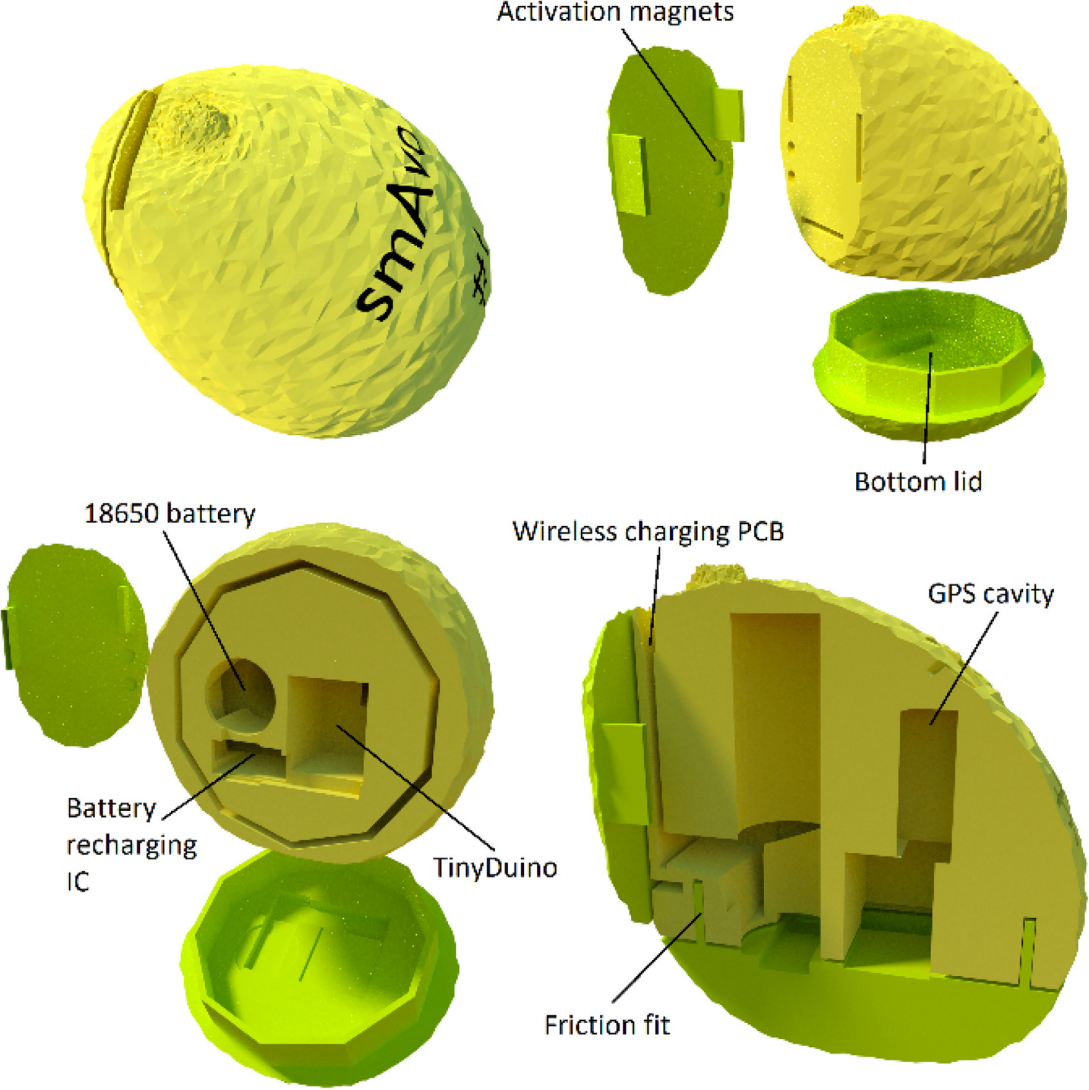
Second prototype of smAvo with integrated wireless charging and magnetic reed switch (Blender).

**Fig. 5:**
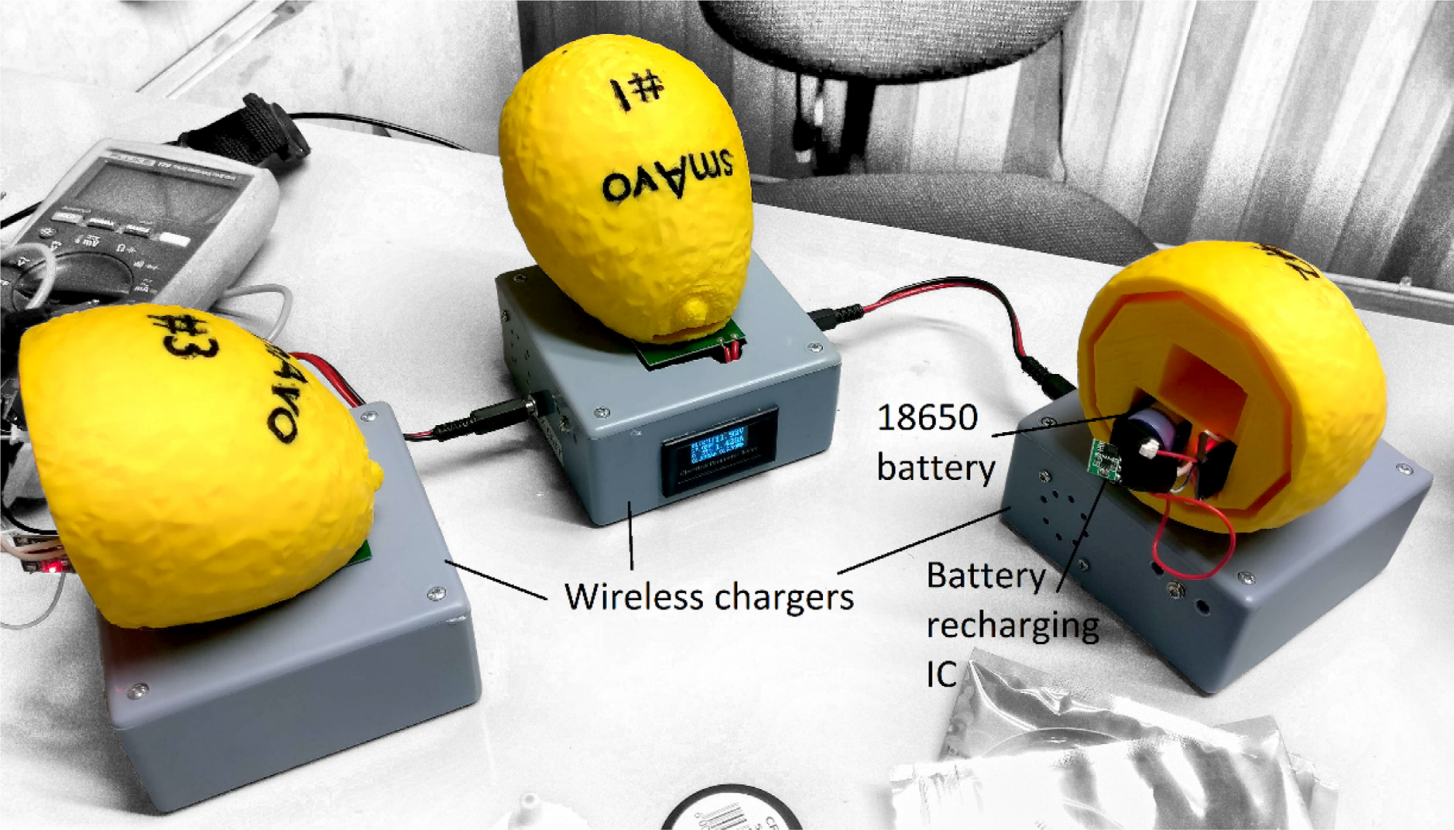
Second prototype in the process of recharging using the wireless chargers.

The first smAvo prototype (Fig. 3) proved that the desired size and volume of a typical avocado could be achieved using a simple design alongside additive manufacturing (3D printing) technologies. This first prototype (371 ml) was used as part of the discussions early in the project, providing an enhanced user experience during the design phase. This was followed thereafter by a second prototype, developed specifically to test the viability of introducing wireless charging capabilities for the smAvo. This feature was combined with the use of an external key to effortlessly power the device on and off while remaining sealed (Fig. 4). A small magnet was glued to the key, which closes a reed switch installed directly beneath the surface of the smAvo body, adjacent to the wireless receiver PCB. A narrow, rectangular slot accommodates the wireless receiver PCB of the charging circuit, with space to accommodate the wires and charging breakout. The wireless receiver PCB was fixed in place with expansion foam. Additionally, a larger 18650-sized battery could be accommodated alongside the battery protection IC and external charging breakout. The rounded interface between the two smAvo sections were replaced by a thicker, 10-sided polygon to prevent free rotation and improve the friction fit between the sections. The wireless emitter PCBs were installed inside small enclosures and powered from a single 12 V power source (Fig. 5).

The initial field tests yielded promising results, but the 3D printed shells encountered a rapid loss of fit resulting from the repeated opening and closing of the enclosure alongside fatigue cracking due to the pressure exerted by the external key. Despite an external layer of cellophane wrap around each smAvo during the field trials, water penetration remained problematic, with water seeping in and out all around the shell. Additionally, the smAvo measured disproportionally large (516 ml) compared to the avocadoes encountered during field testing, which would impact the mesoscale behaviour and resulting sensor measurements. Learning from these experiences, a third and final prototype was developed that could be used for subsequent field tests and instrumentation of the packhouses.

**Fig. 6:**
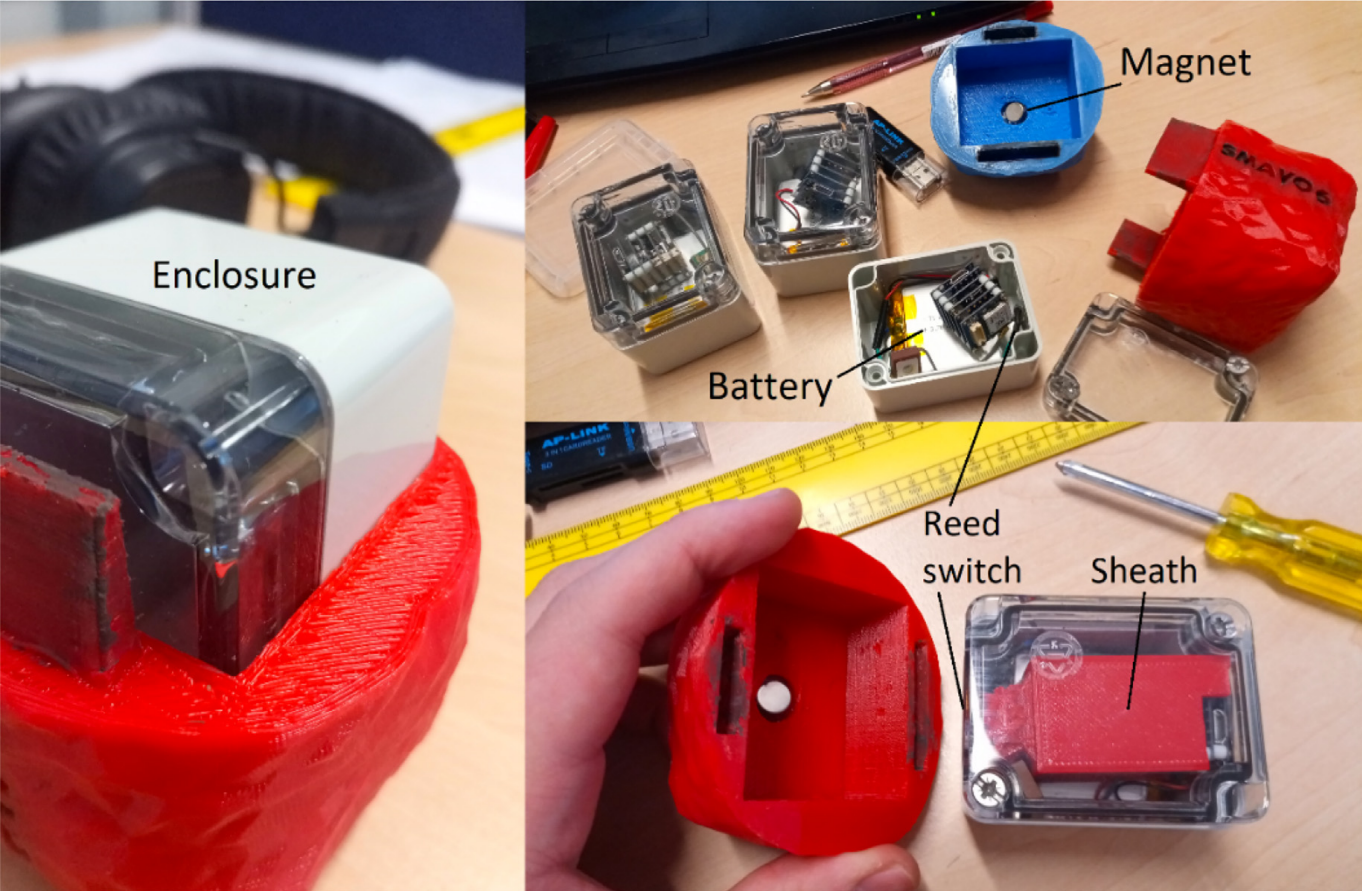
Third and final smAvo prototype illustrating the enclosure, printed shell and embedded magnet.

The third prototype (Fig. 6) was simplified in its design and significantly reduced in volume (down to 319 ml) with a total mass of 198 g. Note that the density for the sealed unit is less than that of water, allowing the instrument to float alongside its biological counterparts. The electronics and battery were installed within the smallest possible IP67 rated enclosure commercially available. As with the second prototype, the sensor platform is powered on and off using a reed switch that is glued to the inside of the waterproof enclosure. A strong magnet in turn is glued to the centre of the shell’s internal surface, reliably powering the sensor platform on and off, without compromising the seal. A secondary magnet is not installed within the opposing shell; significant accelerometer measurement errors were recorded due to the strong magnetic field and proximity of the MEMS accelerometer. Clear markings were added to ensure that the operator installs the shell correctly. The electronic schematic (Fig. 2) illustrates the simplified electrical design. Using Blender - an open source animation and modelling software suite - the shell mesh was moulded around the enclosure until no sharp corners or thin shell sections remained. Small ventilation holes were included at the top of each shell to help both vacate air during closure of the shell and to provide a suction pressure when attempting to remove the shell. The corresponding STL files are available from the data repository (*Models* folder). A rectangular sheath was used to prevent the movement of the TinyDuino stack and maintain a sufficient distance from the activation magnet.

### 5.2 smaTo

**Fig. 7:**
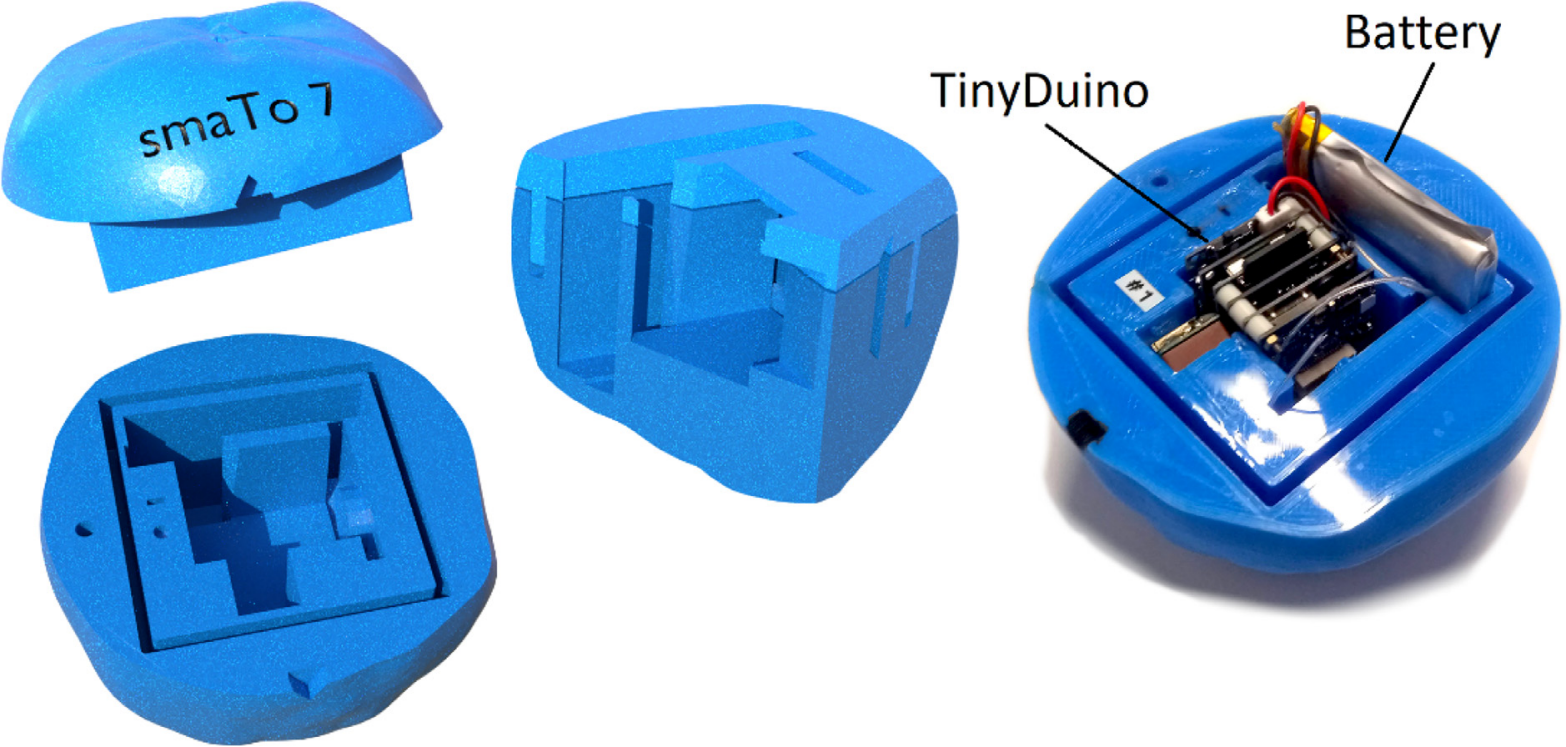
smaTo prototype design illustrating the two hemispheres alongside a 3D printed smaTo with the electronics and battery installed (right).

Following the smAvo, the smaTo was developed soon thereafter (Fig. 7), using a remoulded shell. The smaTo relied primarily on the seal developed by the flexible thermoplastic polyurethane (TPU) filament for waterproofing, with the electronics and battery protected only by the shell. This simplified the design and lowered the cost, with the reed switch mechanism and enclosure eliminated. The total volume of the smaTo was reduced to only 194 ml. Compared to the second smAvo prototype that developed fatigue cracks, no noticeable degradation in the performance of the smaTo have to date been observed. A 0.8% bleeding factor for the lip-and-groove seal provided the best interlock performance, allowing removal of the top half of the shell without significant force to manually toggle the power switch. The corresponding STL files are available from the data repository (*Models* folder). For additional moisture protection, a layer of food-grade cellophane was wrapped around the smaTo and secured in-place with an elastic rubber band. A small piece of cellophane was wrapped around the TinyDuino stack to secure the electronics in place within the shell. Both the switch and SD card are accessible from the front.

### 5.3 3D printing

During the development of the prototypes, various filaments were tested and evaluated for smAvo. The Young’s modulus of avocadoes [28] reduces over time as the fruit ripens, ranging from 480 MPa down to 48 MPa for 0 and 20 days after harvesting respectively [29]. The typical modulus of tomatoes is around 500 MPa [30]. Filaments such as polylactic acid (PLA) are traditionally designed to be as strong as possible, and as a result, exhibit higher Young’s moduli that is reminiscent of biological materials. A firm yet flexible material was required to replicate the characteristics of avocadoes shortly after harvesting.

Various commercially available filaments were trailed during the prototyping phase of the smAvo. For the initial prototypes, a generic PLA filament was used. This is a hard thermoplastic that is easy to print and allows for fine detail in the final product. These initial prototypes worked rather well but were prone to the formation of cracks when subjected to impact forces. For the second prototype, glycol modified polyethylene terephthalate (PETG) was investigated. PETG is also a hard thermoplastic that prints easily, with improved rigidity and elasticity compared to PLA. The final smAvo and smaTo prototypes were printed from a flexible TPU filament that closely resembles a soft rubber. This flexible thermoplastic material, whilst proving more difficult to print, yielded a more durable enclosure suitable for testing applications. The characteristic material properties are summarised in Table 5.

**Table 5:** Summary of the filament material properties for the prototypes.

Material name	Young’s modulus* (MPa)	Izod impact strength** (kJ/m^2^)	Flexural modulus*** (MPa)	Elongation at break* (%)	Price per kg**** (USD)
Polylactic Acid (PLA) [31]	2 347	5.1	3 150	5.2	20.59
Glycol modified polyethylene terephthalate (PETG) [32]	2 100	6.2	2 000	100	25.29
Thermoplastic polyurethane (TPU) [33]	26	34.4	78.7	580	41.17

*ISO527.

**notched at 23 °C, ISO180.

***ISO178.

****From local manufacturer at time of writing.

All the filaments that were tested are widely available in South Africa from commercial retailers. The flexible TPU filament, combined with the friction fit provided by the stiff polycarbonate enclosure, yielded a relatively stiff smAvo that was considered morphologically accurate to the biological specimens. The added flexibility also reduces the susceptibility of the material to fatigue cracking that was experienced with the initial prototypes printed from PLA and PETG filaments. With the small manufacturing tolerance of the polycarbonate enclosure, a filament bleeding factor of 2% was added to the digital model. This ensured a perfect friction between the enclosure and the 3D printed shell (Fig. 6).

**Fig. 8:**
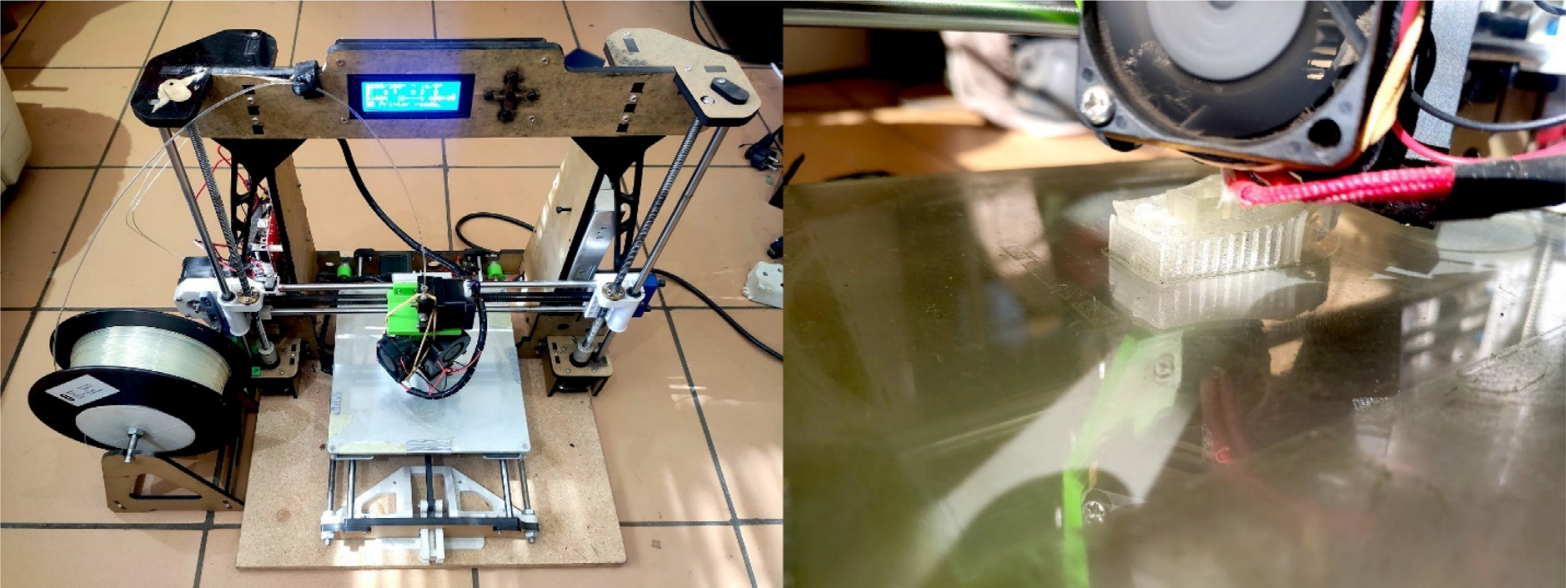
Primary Anet A8 FDM printer used to 3D print prototypes.

All 3D printing activities were executed using a modified Anet A8 FDM printer (Fig. 8). Even though this is typically considered an entry level model, many upgrade modifications are available, including hotends, power supplies and printed components for improved stability, accuracy, and ease of use. Table 6 summarises the printer settings used for each respective material listed in Table 5.

**Table 6:** Optimised 3D printer settings for the selected filament materials.

Material name	Printing temperature (°C)	Layer height (mm)	Printing speed (mm/s)	Infill density (%)
Polylactic Acid (PLA) [31]	200	0.2	60	15
Glycol modified polyethylene terephthalate (PETG) [32]	240	0.2	60	15
Thermoplastic polyurethane (TPU) [33]	210	0.2	40	15

The current implementation of the smAvo requires 180 g of filament to print (including the supports) with an approximate print time of 17 h. Common problems encountered during printing included parts lifting from the print bed and loss of definition of overhangs. These problems were resolved with the addition of carefully positioned support structures underneath overhangs and increasing the print bed’s temperature to 75 °C.

## 6 Operation instructions

Investigation of the postharvest process primarily relies on acceleration measurements to quantify the forces exerted onto the fruit. The addition of auxiliary environmental sensors adds supplementary context and insight to the acceleration history and journey experienced by the sensor platform. The following data channels are available from the current TinyDuino hardware implementation:

1. Tri-axis acceleration (BME280);
2. Sensor platform orientation and rotational velocity (ST LSM9DS1);
3. Barometric air pressure used to infer the location along a pre-determined route (BMP280);
4. Temperature & relative humidity (Si7020-A10);
5. Velocity, heading and geolocation accurate to within 3 m (JF2 TAOS TSL2572), and
6. Light intensity (TAOS TSL2572).

Ideally, all the data would be recorded in parallel for every sensor. Each sensor requires its own specific software libraries, limiting the availability of memory that remains a premium for microcontrollers. Initial firmware development was based on the premise of continuously recording acceleration data with a periodic sampling of auxiliary environmental sensors. Following initial testing, two software scripts were developed using the Arduino IDE that is supported by the TinyDuino architecture. For both scripts, a fixed sampling frequency of 100 Hz was selected for the accelerometer, which proved sufficient for measuring the response of the vehicle’s sprung mass (produce in transit). The firmware and supporting libraries are available from the data repository (*Firmware* folder).

### 6.1 Periodic DAQ script

Tri-axis acceleration measurements (full scale of ± 16-g) are recorded over a period of 30 s and stored in the random access memory (RAM), before writing the data to the non-volatile storage (SD card). Every sample file is stored with the filename corresponding to the Unix time obtained from the RTC. After storing the acceleration data to file, all the other sensors are sampled once and stored in a single log file. The addition of Unix time in the log file allows for easy correlation between the acceleration sample file and the environmental sensor measurements. The cycle requires 30 s to record the acceleration measurements and another 7 s to write the data to storage alongside the other sensor measurements. For extended idle periods, the sensor platform is not required to record high frequency data continuously. A simple, three-tiered activity level was implemented, which is determined by the acceleration’s coefficient of variance (CoV) from the previous sample. If the highest threshold is exceeded, the sensor platform is actively moving in a vehicle or in the packhouse, requiring both high-frequency acceleration and GPS measurements (average current draw of 70 mA). If intermediate activity is recorded, only high-frequency acceleration measurements are stored, saving power through disabling the GPS (average current draw of 14 mA). If no activity is recorded, the sensor platform enters a low-power state, cycling power every three minutes to monitor for new activity (average current draw of 4.5 mA). This active power management continues until either the device is turned off or the battery runs out of charge. These CoV threshold values can be modified within the Arduino sketch.

### 6.2 Continuous DAQ script

For applications that requires uninterrupted accelerometer readings, a continuous stream of data is written to the non-volatile storage. This however limits the accessibility to other environmental sensors, which are sampled only once at the start of the script. At the time of writing, software limitations prevented the simultaneous sampling of environmental sensors together with uninterrupted acceleration sensor measurements. Work is ongoing to resolve these limitations to provide a unified script for optimised performance.

Significant room for improvement exists to optimise the firmware, notably for suspending the processor to a sleep state until activity is recognised by one of the tri-axis accelerometers that consumes minimal power. This reduces the average current consumption of the sensor platform to achieve significantly longer runtimes. Applications that only require monitoring of environmental conditions such as temperature, can be readily configured for such an application to prevent self-heating effects from affecting the measurements. However, this implementation falls outside the scope of the study.

## 7 Validation and characterization

Four field experiments are presented. The first details testing the smAvo’s reliability and general performance by driving along both paved and unpaved roads over a wide speed range. The second and third experiment discusses the smAvo implementation for measuring the both the transportation process from the tree to the packhouse together with an overview of packhouse processing. The fourth experiment represents the validation of the smaTo design in the packhouse prior to more extensive field data undertaken as part of a separate study. The recorded data and selected media files from each experiment is available from the data repository (refer to the *Example data* folder of the data repository).

### 7.1 smAvo – Urban transportation

**Fig. 9:**
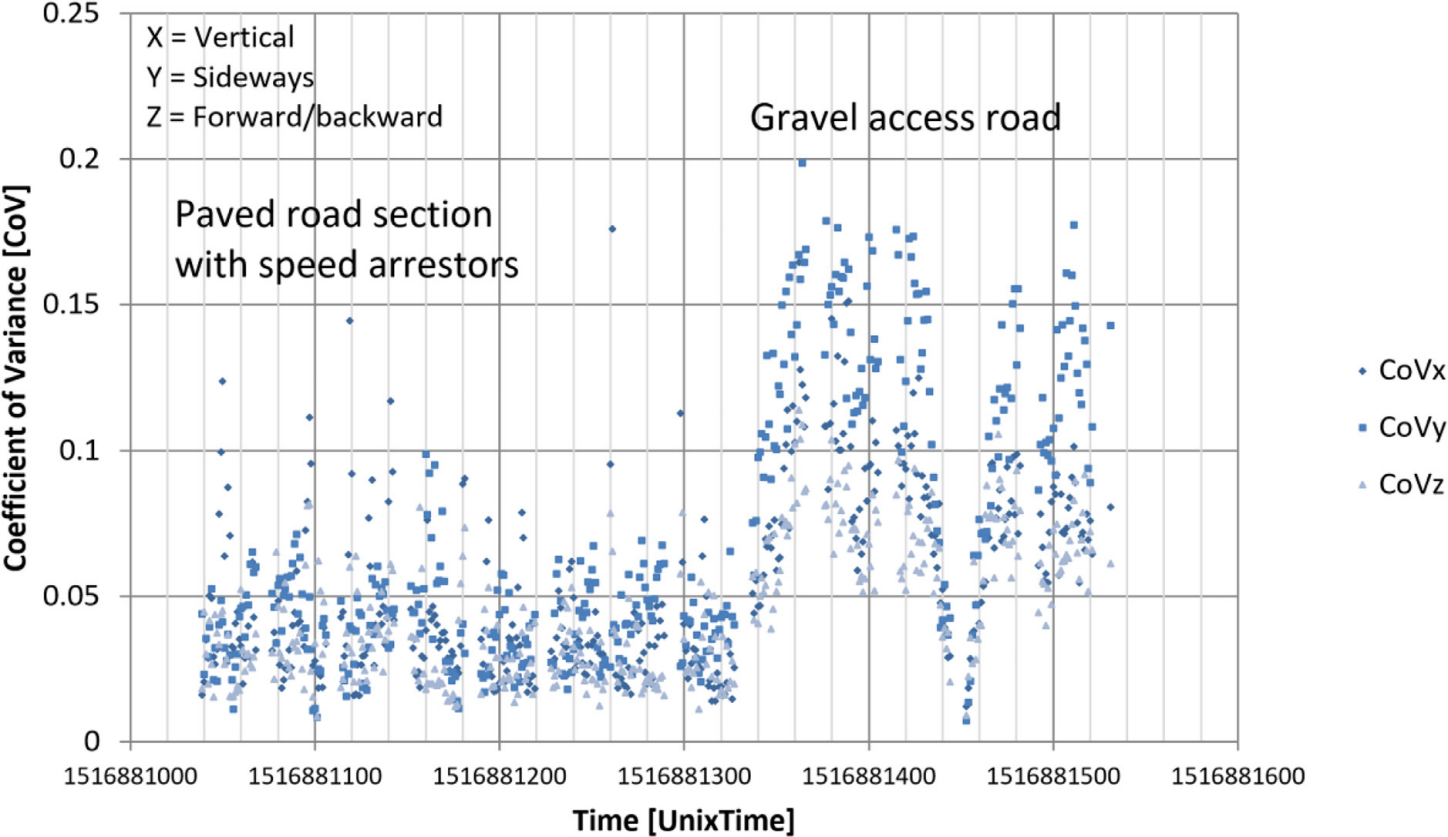
Comparison of acceleration CoV for paved and unpaved section of road.

The second smAvo prototype was used for initial validation and testing of the sensor platform by driving around the Hillcrest campus of the University of Pretoria, in addition to a section of highway from Hatfield to Centurion; this was a combination of both paved and unpaved road sections. The smAvo was placed securely in either the armrest or cup holder of the vehicle while driving. Fig. 9 illustrates the difference between driving on a paved section and a temporary access road constructed from gravel. The coefficient of variance (CoV) metric is commonly applied in transportation engineering as a measure of riding quality. The CoV is calculated from acceleration measurements over a period of one second. For the paved road section, the CoV is significantly smaller than that of the gravel road; severe undulations formed as a result of heavy construction vehicles using the access road for a nearby construction project. As a control, the vehicle was brought to a standstill to validate that the CoV reduces to zero (1516881450).

**Fig. 10:**
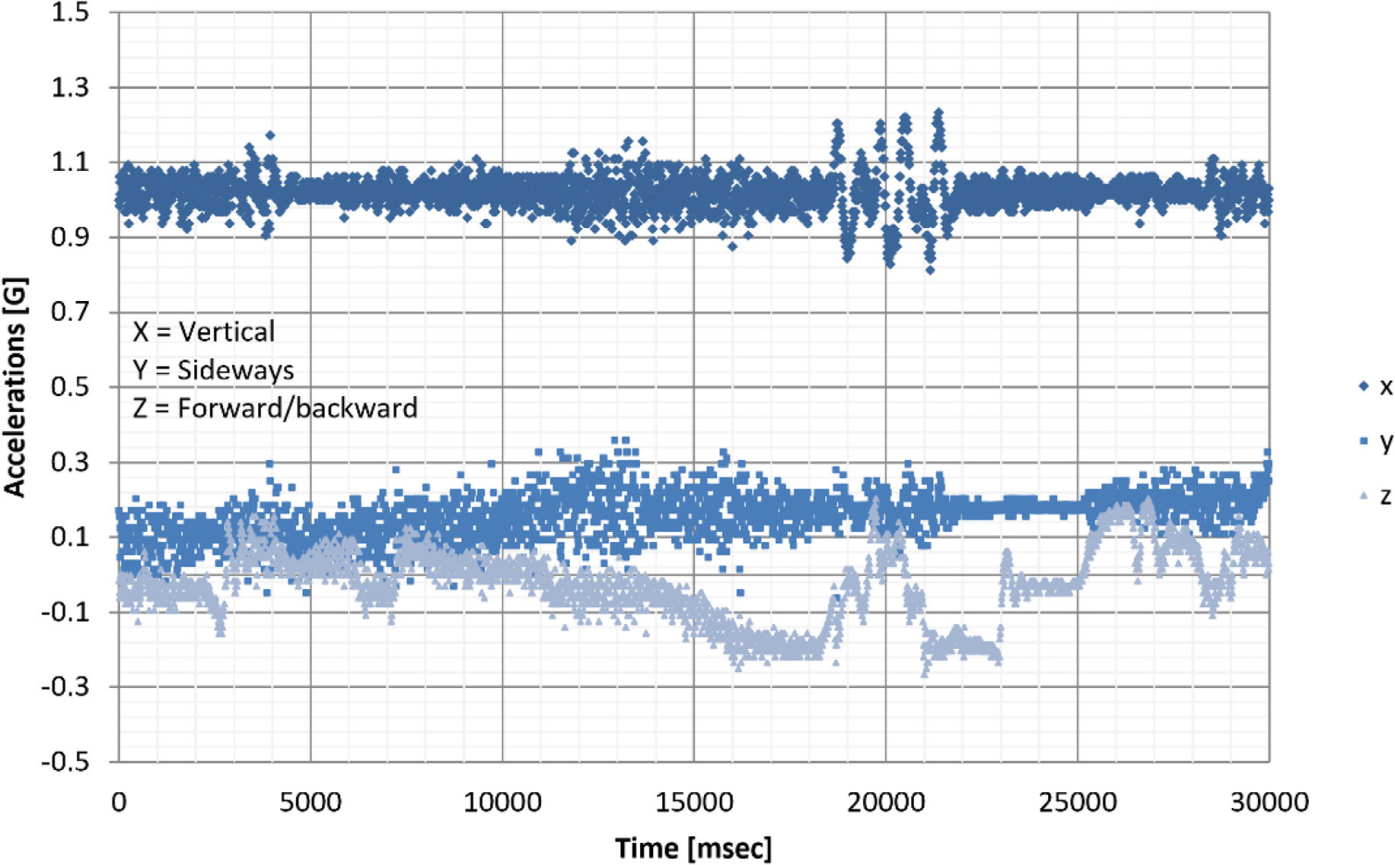
Effect of a speed arrestor in the dynamic vehicle response as measured by the smAvo.

A detailed view of the acceleration measurements (Fig. 10) reveal the vehicle dynamics associated with a speed arrestor. Vertical oscillations (x-axis) of the vehicle’s sprung mass are absorbed by the vehicle’s suspension, dampening rapidly. The braking and acceleration of the vehicle, before and after the arrestor respectively, are also clearly visible. Lateral accelerations of the smAvo are large unaffected (y-axis).

**Fig. 11:**
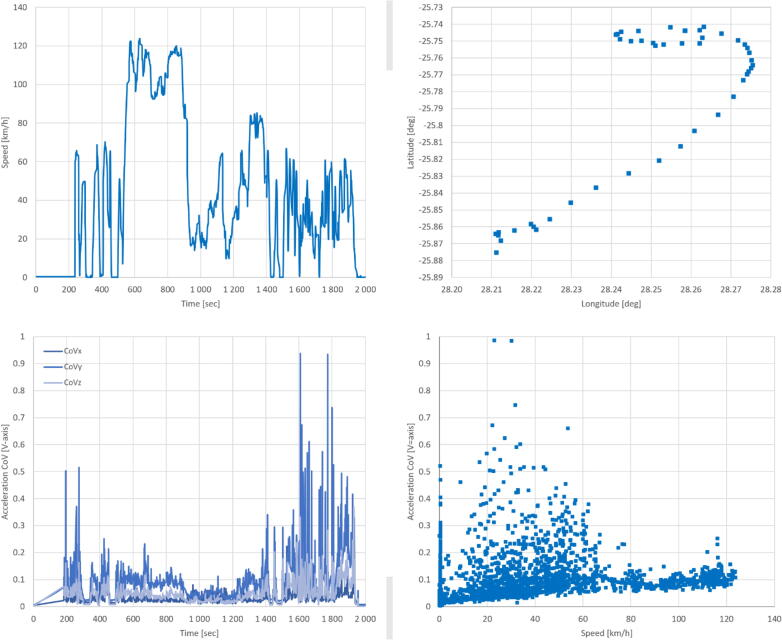
GPS information recorded for a longer journey with varying road conditions.

For journeys over a longer period of time, where the vehicle travelled along both city streets and the freeway, more complex responses are observed (Fig. 11). The velocity (Fig. 11, top-left) recorded by the smAvo’s GPS (Fig. 11, top-right) matched the vehicle’s velocity as observed by the driver during the journey, spanning a total distance of approximately 30 km. Graphing the acceleration CoV (Fig. 11, bottom-left) measurements as a function of the vehicle’s velocity (Fig. 11, bottom-right) reveals that the freeway is associated with less acceleration and braking action, compared to the slower city traffic. The acceleration CoV peaks noticeably peaks for velocities approaching zero, as this is associated with the vehicle either coming to a complete stop or accelerating from an idle position. The GPS signal strength was sensitive to any obstructions around the device, likely due to the small footprint of the antenna. Due to the drift of the RTC of each smAvo, synchronisation for all the instruments is required approximately every three months. With the sensor platform reliability validated, full-scale field trials were conducted outside the town of Tzaneen in the Limpopo province for both the smAvo and smaTo, in partnership with ZZ2.

### 7.2 smAvo – Farm to packhouse

**Fig. 12:**
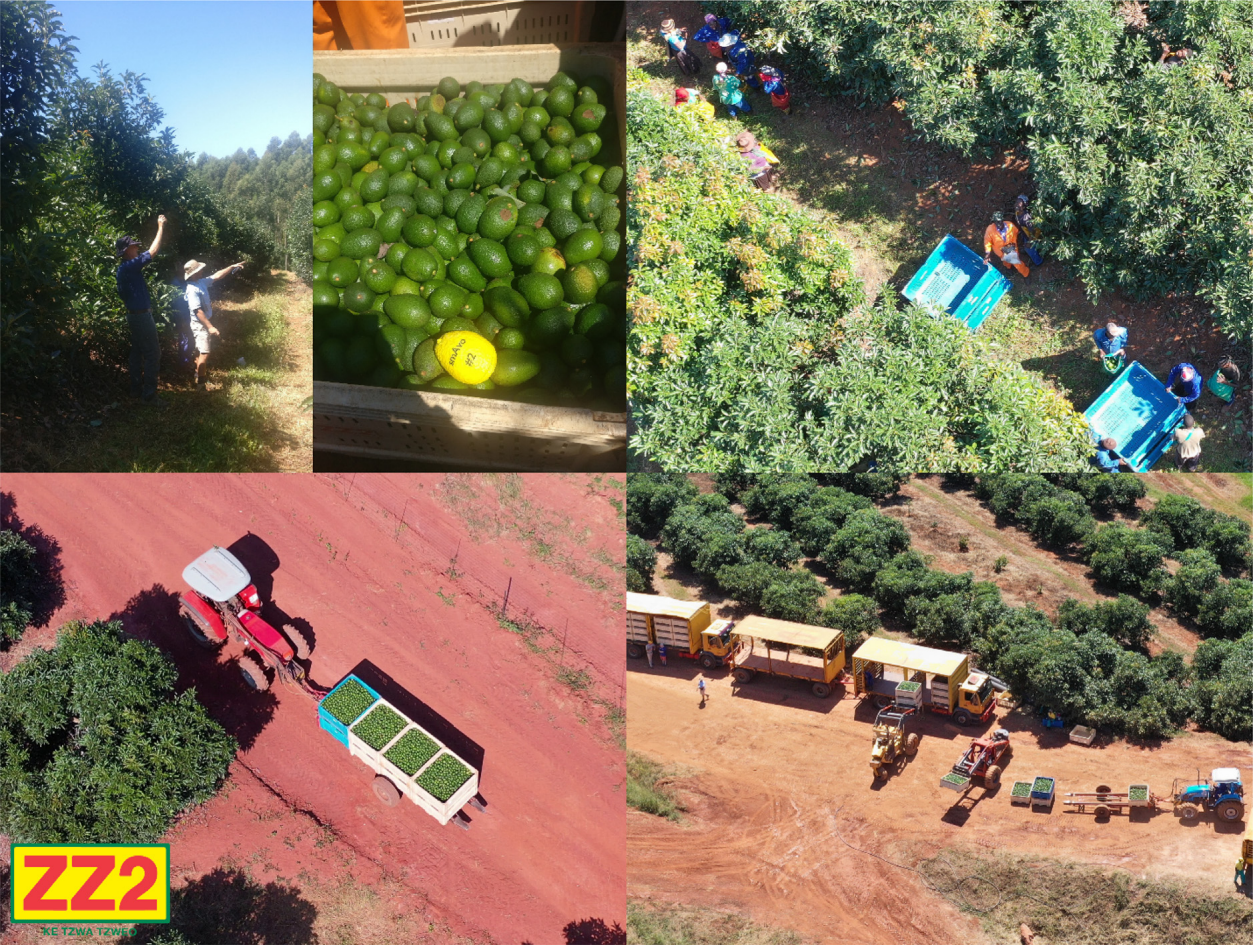
Picking and transportation of the avocadoes (top row). The avocadoes are collected in bins and transported (bottom-left) to the dansvloer (bottom-right) for the final journey to the packhouse.

The results presented for smAvo illustrate a combination of data collected from two separate trials, with the farm-to-packhouse measurements recorded by the second smAvo prototype, and the packhouse measurements recorded by the third smAvo prototype. The farm-to packhouse phase aimed to distinguish the primary modes of transportation. A detailed checklist was developed to ensure repeatability of the measures (refer to the *Example data* folder of the data repository). Fig. 12 illustrates the distinct phases, from “picking” the smAvo and tipping the bag of fruit into the larger bins, collection of the bins by a tractor, and transportation of the bins to a centralised location, referred to as the *dansvloer* (dancefloor). The bins were loaded onto larger interlink trucks which travelled to the centralized packhouse for processing. The entire harvesting process is engineered to minimise the amount of time the avocadoes are exposed to uncontrolled environmental conditions. The storage temperature of the avocadoes is lowered to between 3 °C and 6 °C, slowing the ripening process of the fruit. Even with a relatively small battery capacity of 1 100 mAh (for the third smAvo prototype), the smAvo and smaTo are expected to provide continuous operation for a 15-hour period. This enables the recording of a complete journey undertaken on the road between Tzaneen in the Limpopo province and Pretoria in the Gauteng province.

**Fig. 13:**
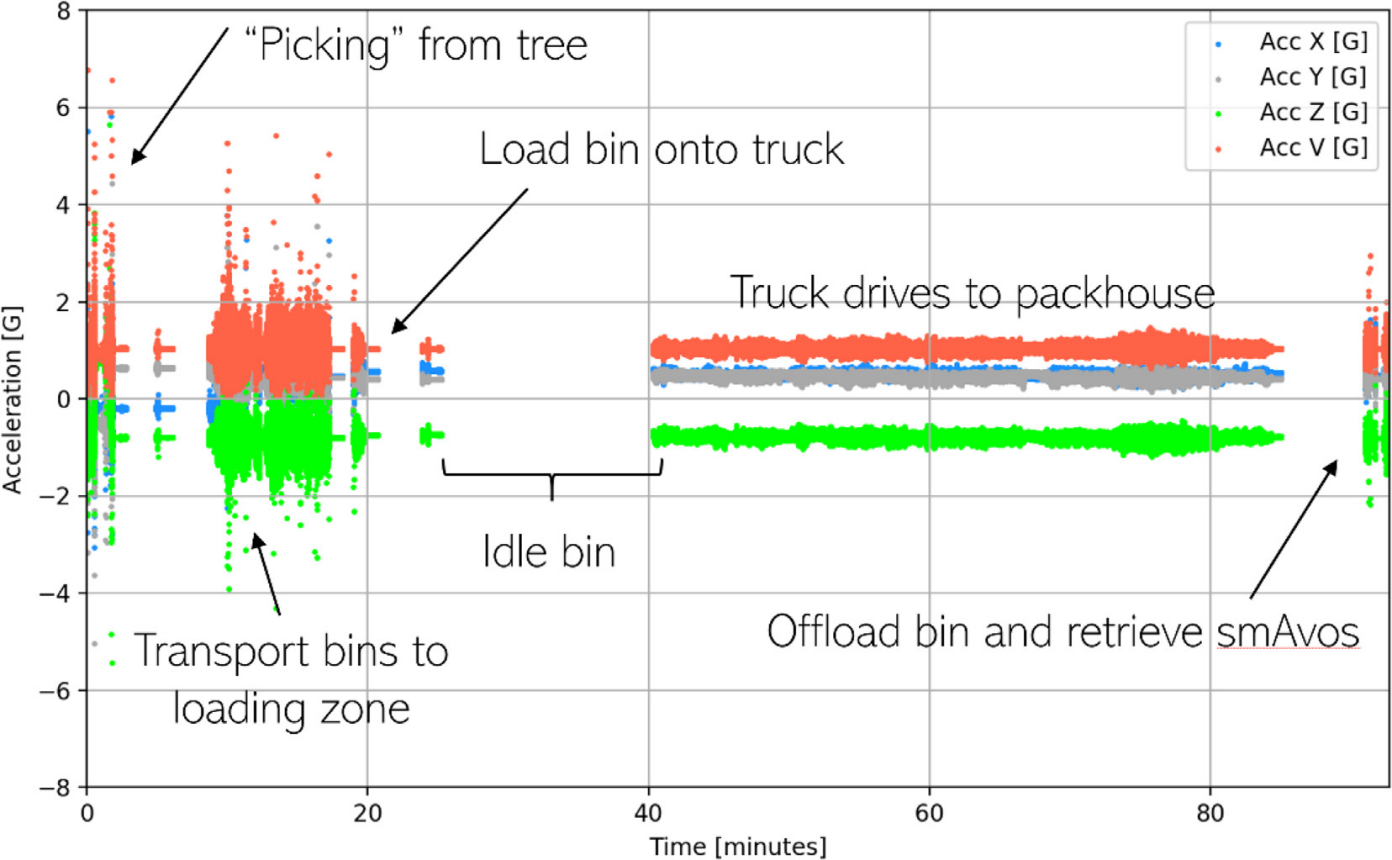
Acceleration history for the tree-to-packhouse field trial.

The acceleration measurements from the farm-to-packhouse experiment are illustrated in Fig. 13, spanning over a period of 90  min and a total distance of 32.4 km. Similar to the graphical illustration (Fig. 12), the distinct transportation phases can be readily identified. The highest intensity acceleration measurements at beginning are associated with the pick-and-carry process by the farm workers. High intensity accelerations are associated with the tractor that moves the bins to the *dansvloer* at the 15-minute mark. It was noted that the tractor did not have any form of suspension for its trailer that carried the bins. The bin with the smAvo was loaded onto the truck at the 23-minute mark, followed by a 17-minute rest period while the remainder of the truck was loaded to capacity. The truck drove a total of 42 min, the majority of which covered paved roads, followed by a short section of unpaved road near the end of the drive. The end of the test (90-minute mark) was characterised by a forklift offloading the bins from the truck onto the packhouse floor, followed by manually retrieving powering off the smAvos.

**Fig. 14:**
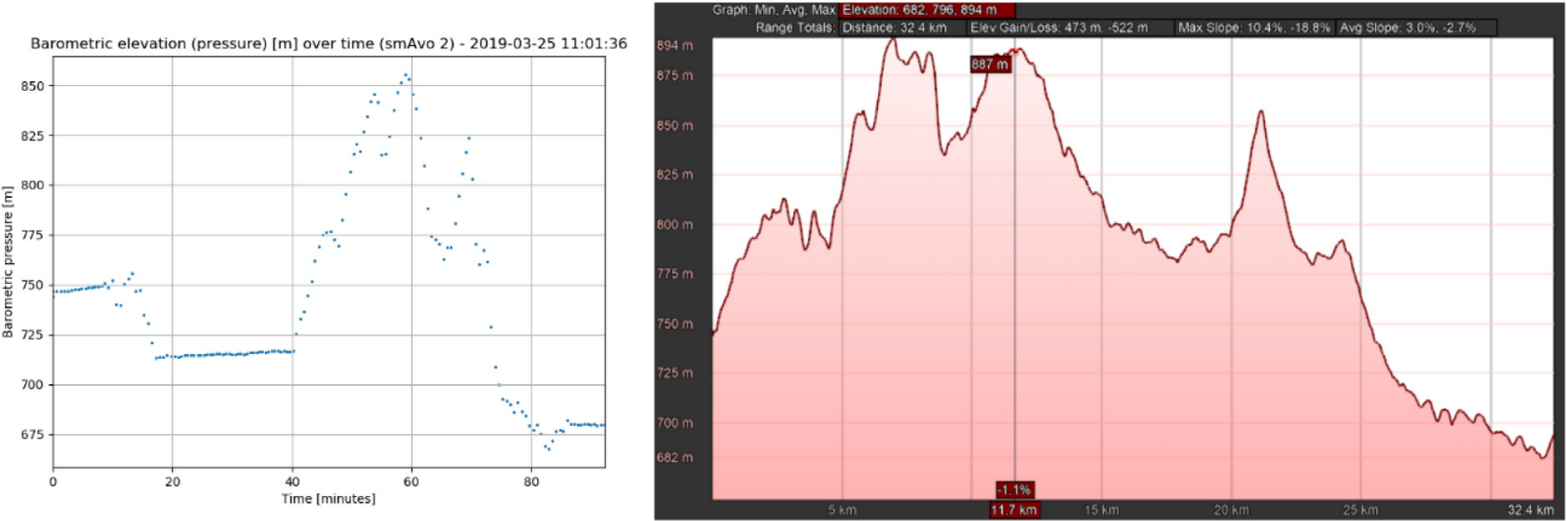
Comparison between the measured barometric air pressure measured by smAvo (left) and an elevation profile obtained from Google for the same route (right).

As a result of the dense packing of the avocadoes within the bins, the smAvos only recorded sparse GPS information at the start and end of the experiments. However, comparing the barometric air pressure readings from the smAvo to that of the corresponding elevation profile generated using Google Earth, a clear correlation can be observed (Fig. 14). Note that the smAvo measurements are represented as a function of time and the elevation profile from Google Earth is represented as a function of distance. The peaks and valleys of the measured air pressure can be traced exactly along the elevation path, and thus the corresponding geolocation, provided that the travel route is predefined. The average speed of the loaded truck was reduced to approximately half for positive road elevations compared to descending roads, hence the distortion of the time-domain graph along the x-axis.

### 7.3 smAvo – Packhouse characterisation

**Fig. 15:**
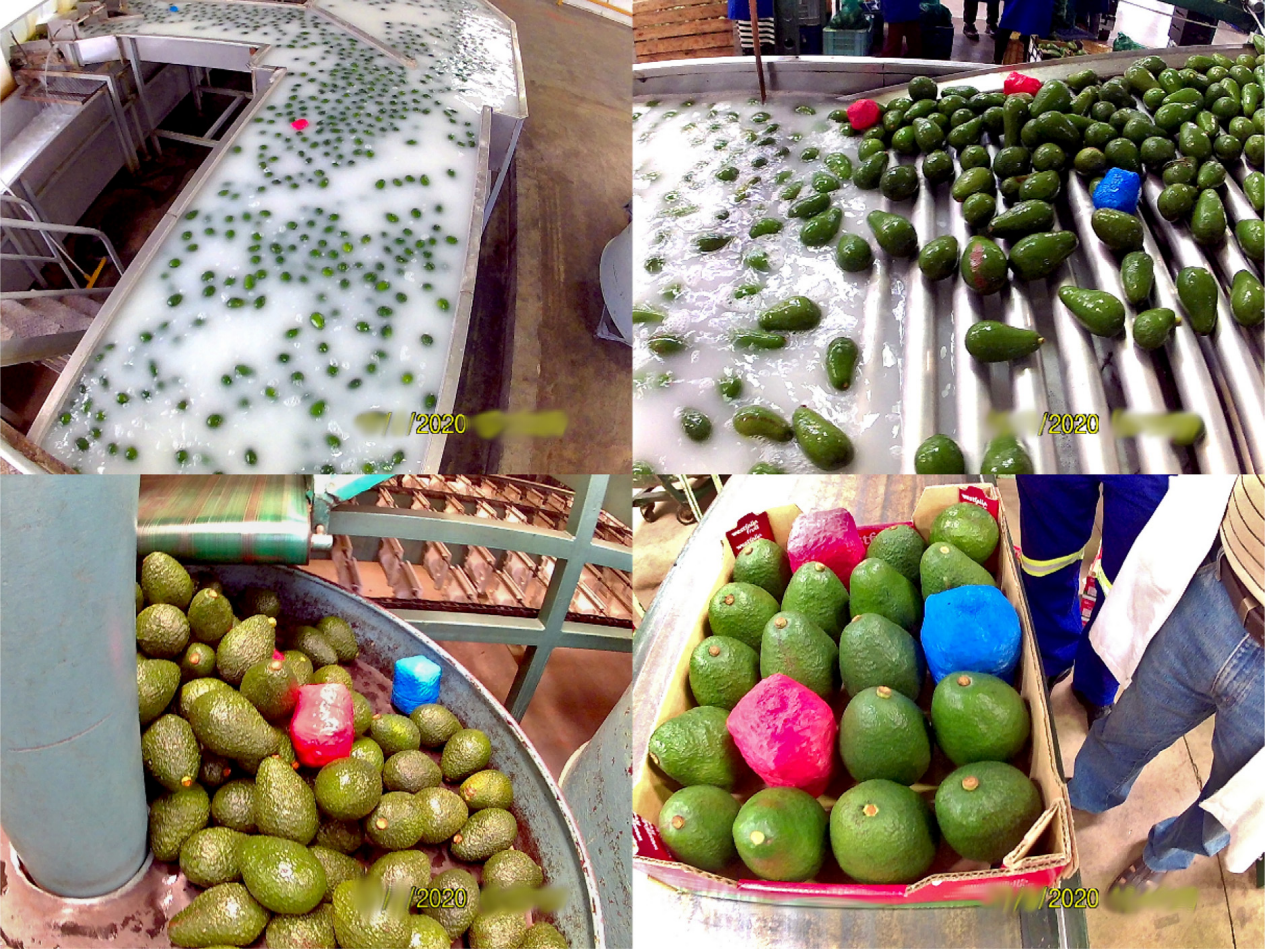
Avocado packhouse illustrating the postharvest treatment (top-left), drying (top-right), sorting (bottom-left) and packing (bottom-right) processes.

**Fig. 16:**
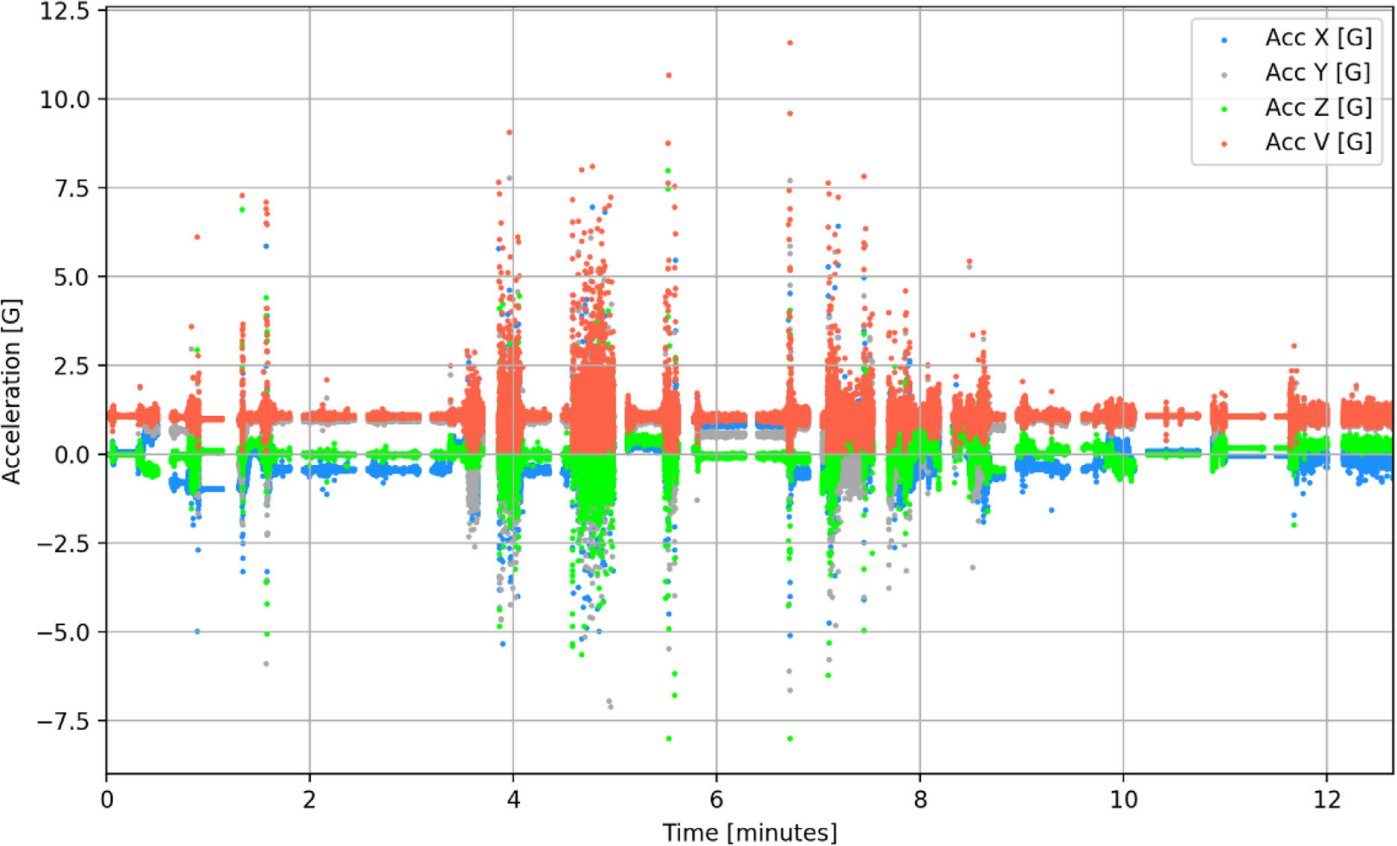
Acceleration history of a smAvo (third prototype) processed in a mechanized packhouse facility.

Based on the research carried out for this first trial, a larger testing program was established to measure all the avocado packhouses in the Limpopo and KwaZulu-Natal provinces. The successful introduction of smAvo enables significantly faster and lower cost data acquisition than what was possible before. A single trained technician can instrument three to four packhouses per day. Each packhouse is sampled twice to double the sample size obtained by the four smAvos currently in service. Fig. 15 illustrates the typical packhouse environment that encompasses screening, washing, fungicide application, drying, sorting, and packaging. Fig. 16 illustrates the acceleration history of one of the smAvos for a specific packhouse. Evidently, a complex acceleration history was recorded by the smAvo, with various machines and processes influencing both the amplitude and duration of the accelerations experienced. Significant peak-to-peak accelerations approaching 20-g were primarily the result of the fruit falling a short distance from one conveyer to another. The vector acceleration (denoted as *Acc V*) was defined as the true acceleration, combining the orthogonal acceleration components (*Acc X* , *Acc Y* and *Acc Z*) using the Pythagorean expression.

### 7.4 smaTo – Packhouse characterisation

**Fig. 17:**
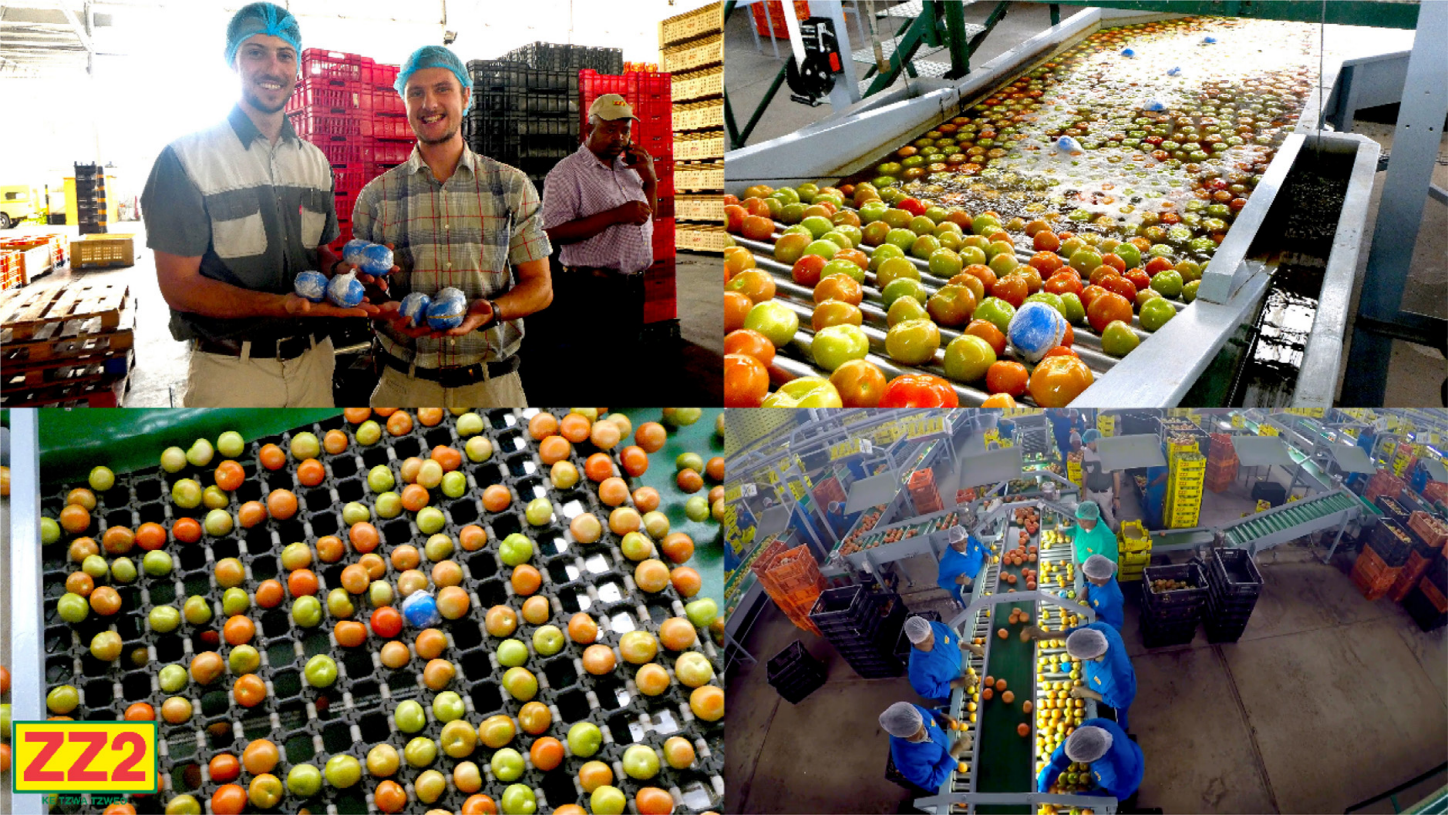
smaTo validation test at the ZZ2 packhouse. smaTo assistants for the day: Hendus Janse van Rensburg (left) and Frannes Joubert (right).

**Fig. 18:**
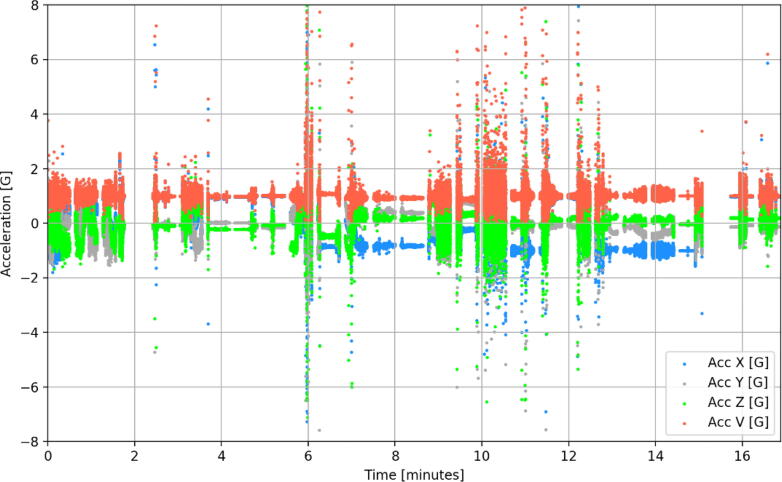
Acceleration history of a smaTo processed in a mechanised packhouse facility.

Following the successful field trials of smAvo, smaTo’s performance was validated in a similar packhouse facility to that of the avocadoes (Fig. 17). The blue coloured filament assists with identifying the smaTos among the many thousands of tomatoes processed every hour. A similar processing procedure to avocado was followed for the tomatoes, including washing, drying, sorting, and packaging. After powering on the instruments, a single layer of cellophane plastic enveloped the smaTo with a rubber band securing the film in-place, providing additional resistance to moisture. The complex acceleration history of the smaTo (Fig. 18) is reminiscent of the smAvo (Fig. 16), both in amplitude, periodicity, and duration. The acquisition and in-depth analysis of packhouse data using smAvo and smaTo is ongoing.

From the experience and experimental results obtained during the development and validation of smAvo and smaTo, the capabilities and limitations of the hardware are summarised:

1. The experimental results illustrated reliable instrumentation performance for all the field trials. The tri-axis, MEMS-based accelerometer measured the quantitative, mesoscale response of a single fruit in a process typically described and characterised by stochastic behaviour;
2. Additive manufacturing (3D printing) proved invaluable in designing and replicating a synthetic fruit, with comparable morphological characteristics to its biological counterpart. Long-term performance of the material subjected to chemical handling is yet to be established;
3. The sampling frequency (100 Hz) and full-scale (±16-g) of the accelerometer are adequate to record sensor measurements continuously with the required definition;
4. Auxiliary GPS and barometric air pressure sensors can be utilised to accurately geo-locate the sensor platform either directly or indirectly, respectively;
5. The software of the TinyDuino can be improved to record acceleration data continuously instead of 30 s increments together with the auxiliary environmental sensors;
6. The implementation of high-capacity lithium batteries and a power efficient micro-controller are suitable to record data throughout the entire transportation process, from farm-to-the-fork, over the span of days or even weeks;
7. Depending on the intended application, waterproofing and dust resistance can form part of the instrumentation design without compromising the quality of the sensor measurements, and
8. Data retrieval and processing remains labour intensive. The addition of automatic, wireless data retrieval and edge computing technologies should be introduced for commercial use of such devices to realise the cost effective optimisation of postharvest processing.

Investigation of the primary variables affecting the magnitude of postharvest stress experienced by avocadoes in packhouse environments is currently underway (smAvo), in addition to the effect of extended transportation routes by truck (smaTo). A total of 20 packhouses located across 3 provinces have been instrumented with smAvo during the 2020 avocado season, the results of which will soon be published.

## Funding

This work was supported by the South African Avocado Growers Association (Project 33/19) (South Africa), the Tomato Producers Organization (Project 29/19) (South Africa), the DST Post Harvest Innovation Programme (Projects 29/19 and 33/19) (South Africa) the 10.13039/501100001321National Research Foundation (Project 119797) (South Africa) and ZZ2 (South Africa).

## CRediT authorship contribution statement

**André Broekman:** Conceptualization, Methodology, Software, Validation, Investigation, Formal analysis, Data curation, Writing - original draft. **Wynand JvdM Steyn:** Funding acquisition, Resources, Project administration, Writing - review & editing, Supervision, Funding acquisition. **Johannes LP Steyn:** Methodology, Investigation. **Malick Bill:** Supervision, Project administration, Writing - review & editing. **Lise Korsten:** Supervision, Project administration, Writing - review & editing.

## Declaration of Competing Interest

The authors declare that they have no known competing financial interests or personal relationships that could have appeared to influence the work reported in this paper.
